# The Efficacy of Hesperidin in the Reduction of Atherosclerosis in ApoE^−^^/−^ Mice and Its Possible Mechanism of Action

**DOI:** 10.3390/foods14203560

**Published:** 2025-10-19

**Authors:** Qi Wang, Xiaoxia Huang, Mengyao Zhang, Shangyuan Sang, Linrong Fang, Ruilin Zhang, Silei Xia, Yanan Liu

**Affiliations:** 1 Zhejiang-Malaysia Joint Research Laboratory for Agricultural Product Processing and Nutrition, Provincial Key Laboratory of Food Microbiology and Nutritional Health, Department of Food Science and Engineering, Ningbo University, Ningbo 315800, China; 16639670422@163.com (Q.W.); 18757477952@163.com (X.H.); z2154558544@163.com (M.Z.); sangshangyuan@nbu.edu.cn (S.S.); 15586611584@163.com (L.F.); 2 Key Laboratory of Detection and Risk Prevention of Key Hazardous Materials in Food, China General Chamber of Commerce, Ningbo Key Laboratory of Detection, Control, and Early Warning of Key Hazardous Materials in Food, Ningbo Academy of Product and Food Quality Inspection (Ningbo Fibre Inspection Institute), Ningbo 315048, China; 3 Central Laboratory, Shenzhen University General Hospital, Shenzhen 518055, China; ruilin_zhang92@163.com; 4 Guangdong Key Laboratory for Biomedical Measurements and Ultrasound Imaging, Department of Biomedical Engineering, School of Medicine, Shenzhen University, Shenzhen 518060, China; 5 College of Marine and Biology Engineering, Yancheng Institute of Technology, Yancheng 224051, China; 6 College of Food Science and Technology, Nanjing Agricultural University, Nanjing 210095, China

**Keywords:** hesperidin, atherosclerosis, gut microbiota, branched-chain amino acid

## Abstract

Atherosclerosis (AS) currently lacks fully effective treatments. This study investigated the natural compound hesperidin as a potential therapy. Apolipoprotein E knockout (ApoE^−/−^) mice were used as a model of atherosclerosis; we found that hesperidin treatment improved physiological and metabolic health, reduced plaque formation, and decreased systemic inflammation and oxidative stress. Hesperidin also reshaped gut microbiota, increasing beneficial bacteria (*Verrucomicrobia* and *Bacteroidota*) and significantly lowering fecal levels of branched-chain amino acids (BCAAs: valine, leucine, and isoleucine) by 27.4%, 50.1%, and 40.8%, respectively. These changes were linked to specific microbial shifts. We conclude that hesperidin alleviates atherosclerosis likely by modulating the gut microbiota–BCAA–host axis, identifying it as a promising dietary intervention or therapeutic agent.

## 1. Introduction

Atherosclerosis (AS) is a prevalent cardiovascular disease characterized by arterial lipid accumulation, fibrous tissue proliferation, and calcification [1] and is linked to high morbidity and mortality worldwide, including most myocardial infarctions and strokes, as well as disabling peripheral arterial disease [2]. Although often asymptomatic initially, advanced plaques can obstruct blood flow, leading to ischemia and serious complications such as coronary artery disease [3]. Current treatments, such as lipid-lowering drugs, lifestyle changes, and surgery, have limited effectiveness and are often associated with side effects. Thus, developing safer and more effective strategies is urgently needed.

Natural compounds like hesperidin have recently shown potential in combating cardiovascular diseases [4]. Hesperidin, a flavonoid abundant in citrus peels [5], exhibits anti-inflammatory, antioxidant, and lipid-regulating properties [6]. It has demonstrated beneficial effects in improving lipid metabolism and atherosclerosis in preclinical models, as well as antihypertensive and antioxidant Activities [7,8]. However, its full mechanism of action remains unclear, particularly whether it modulates the “gut-axis” regulatory network—a rising focus in life sciences.

Disturbed branched-chain amino acid (BCAA) metabolism contributes significantly to atherosclerosis [9]. The gut microbiota acts as a key upstream regulator: certain bacteria (*Bacteroides*, *Prevotella*) metabolize dietary BCAAs, while microbial dysbiosis can impair BCAA breakdown, leading to elevated circulating BCAA levels [10]. Excess BCAAs promote insulin resistance, inflammation, endothelial dysfunction, and macrophage activation—processes that accelerate AS [11,12]. Therefore, targeting gut microbiota to regulate BCAA metabolism represents a promising therapeutic approach.

Based on this, we hypothesize that hesperidin may attenuate atherosclerosis by remodeling the gut microbiota and enhancing bacterial BCAA degradation, thereby reducing systemic BCAA levels. To rigorously test this hypothesis in vivo, the ApoE knockout mouse model is essential, as it spontaneously develops human-like hyperlipidemia and atherosclerosis, providing a robust platform to study hesperidin’s modulation of gut microbiota and BCAA metabolism in AS, this study aims to evaluate the anti-atherosclerotic effects of hesperidin and elucidate its mechanisms via the “gut microbiota–metabolite–host” axis, providing new insights into targeting the gut microbiome for AS prevention and treatment.

## 2. Materials and Methods

### 2.1. Chemicals

Hesperidin (purity > 95%) was purchased from Shanghai Yuanye Biological Co., Ltd. (Shanghai, China), atorvastatin (purity > 99%) was purchased from Shanghai McLean Biochemical Co., Ltd. (Shanghai, China), sodium carboxymethyl cellulose (CMC-Na) was purchased from Shanghai McLean Biochemical Co., Ltd. (Shanghai, China), and all the reagent kits were purchased from Nanjing Jiancheng Bioengineering Co., Ltd. (Nanjing, China). The limits of detection were below 5 mg/dL for TG, 10 mg/dL for TC, and 3 mg/dL for both HDL-C and LDL-C. For the enzyme assays, the detection limits were typically below 3 U/L for both AST and ALT. The precision profiles were excellent; at medically relevant concentrations, the intra-assay coefficients of variation (CVs) were generally ≤2.5%, and the inter-assay CVs were ≤4.0%. All reagents were analytically pure.

### 2.2. Animal Procedures and Treatments

A total of 20 male C57 mice and 30 male ApoE^−/−^ mice, all aged 6–8 weeks and weighing 20 ± 2 g, were used in this experiment [13]. The mice were obtained from Jiangsu Jicui Pharmachem Biological Co., Ltd. (Nanjing, China) and were housed at the Animal Experiment Centre of Ningbo University (Ningbo, China). All mice were housed in standard cages at 22–24 °C, 50–55% relative humidity, and a 12-h light-dark cycle. The animal experiments were conducted following the Chinese National Standardization Administration Committee’s guidelines for the ethical use of experimental animals and were approved by the Laboratory Animal Management and Ethics Committee of Ningbo University with the number 12,621. The study utilized SPF-grade mice purchased from Jiangsu GemPharmatech Co., Ltd. (Nanjing, China), which were obtained from an independent source. After a one-week acclimatization period, all animals were randomly assigned to the various treatment groups. Due to the different diets used in the experiment, blinding was not feasible for the duration of the study. Blinding was implemented for the measurement of atherosclerosis.

Prior to the start of the hesperidin treatment experiments, all mice were acclimatized and fed for one week, after which they were weighed. To ensure a balanced weight for each group of mice, overweight and lean mice were eliminated after being weighed. C57 mice were then divided into two groups (*n* = 9): a normal control group (CD) and a hesperidin-treated control group (CD + Hes), adopt a normal diet. ApoE^−/−^ mice were divided into three groups (*n* = 9): an atherosclerosis control group (HFD), a hesperidin-treated group (HFD + Hes), and a positive drug (Atorvastatin) control group (HFD + AT), adopt a high-fat diet. The normal diet and the high-fat/high-cholesterol diet were purchased from Jiangsu Synergy Pharmaceuticals Bioengineering Co., Ltd. (Nanjing, China). Throughout the study, the CD and HFD control groups were orally administered an equal volume of 0.5% CMC-Na solution by gavage. According to the literature, the administration dose of hesperidin [14] was 150 mg/kg/d (corresponding to a human equivalent dose of ~12.2 mg/kg), dissolved in 0.5% CMC-Na and administered orally by gavage. Similarly, atorvastatin was dissolved in 0.5% CMC-Na for oral gavage. All treatments were continued concurrently for a period of 12 weeks.

Mice were weighed and recorded once a week. One week before the end of the experiment, freshly collected faecal samples were collected for later analysis and stored frozen at −80 °C. After 12 h of fasting, all mice were anaesthetised with isoflurane and then decapitated and killed. The eyeballs were removed to collect blood from the orbital region, followed by centrifugation (4 °C, 6000× *g*, 10 min). Hearts and aortic structures were removed and placed in 4% paraformaldehyde for fixation, and liver tissues were collected and rapidly frozen in liquid nitrogen. Finally, the mouse carcasses were temporarily stored at the Animal Entity Recovery Centre.

### 2.3. Analysis of Biochemical Indicators

Triglycerides (TG), cholesterol (TC), high-density lipoprotein cholesterol (HDL-C), low-density lipoprotein cholesterol (LDL-C), aspartate aminotransferase (AST), and alanine aminotransferase (ALT) in serum samples were assayed using the appropriate kits and a fully automated biochemistry analyzer (08IL IHOVLIN, Fukui, Japan). Liver samples were also analyzed for AST and ALT. About 100 mg of liver from each group was weighed in a mortar, and saline was added in the ratio of 1:6. After thorough grinding, the samples were centrifuged at 3000× *g* for 10 min and the supernatant was separated for further assay. interleukin-6 (IL-6), tumor necrosis factor-alpha (TNF-α), Superoxide dismutase (SOD), total bile acids (TBA) and oxidized low-density lipoprotein (ox-LDL) cytokine levels in liver were determined by ELISA [15]. Briefly, a standard curve is established using the standard reagents in the kit. The solution to be measured is then reacted with the enzyme standard reagent and the color developer is added. Finally, a termination solution is added and the assay is then labelled with the enzyme and compared to the standard curve.

### 2.4. Histopathology

After excision, the entire heart aorta was carefully cleaned of peripheral adipose tissue and fixed in 4% paraformaldehyde for 24 h. The tissues were dehydrated through an ethanol gradient, embedded in paraffin, and sectioned at 4–6 μm thickness. Sections were deparaffinized and stained with hematoxylin and eosin (H&E) following standard protocols. Imaging was performed under a light microscope (Nikon Eclipse CI, Tokyo, Japan) at 100× magnification in a blinded manner. For lipid plaque visualization, aortic cryosections were stained with Oil Red O. All histological processing and staining were performed by Wuhan Baiqiandu Biotechnology Co., Ltd. (Wuhan, China). Plaque areas were quantified using Image J 1.x software.

### 2.5. Quantitative 16S rRNA Sequencing

Fecal samples were collected after 11 weeks of intervention, immediately frozen in liquid nitrogen, and sent to LC-Bio Technology Co., Ltd. (Hangzhou, China) for 16S rRNA sequencing. Briefly, microbial genomic DNA was extracted, and the V3–V4 hypervariable regions of the 16S rRNA gene were amplified. Sequencing was performed on the IlluminaNovaSeq 6000 platform (San Diego, CA, USA). Bioinformatic analysis including ASV clustering, taxonomic annotation, and diversity assessment was conducted by the company.

### 2.6. Determination of BCAA Content

BCAA levels in fecal samples were analyzed using liquid chromatography-tandem mass spectrometry (LC-MS/MS). After sample homogenization and derivatization, extracts were filtered and sent to Shanghai Metabio Biomedical Technology Co., Ltd. (Shanghai, China) for LC-MS/MS analysis using an SCIEX QTRAP 6500+ system (SCIEX, Framingham, MA, USA) equipped with an AdvanceBio MS Spent Media column. Mass spectrometry data were collected using the default parameters of the AB Sciex OS (SCIEX OS 1.4.x) (SCIEX, Framingham, MA, USA) quantitative software, which automatically identifies and integrates the ion fragments and assists with manual checking.

### 2.7. Data Analysis

The data and statistical analysis complys with the recommendations on experimental design and analysis in pharmacology [16]. Data was expressed as means ± SD and analyzed by using GraphPad Prism 8.0 software (San Diego, CA, USA) and SPSS 25.0. The normality of distribution was assessed using the Shapiro–Wilk test, and homogeneity of variances was confirmed with Brown-Forsythe test. Upon verification of parametric test assumptions, One-way ANOVA for comparisons between multiple groups, with a post hoc Tukey’s test to control for Type I error in multiple comparisons. The value of *p* < 0.05 was considered statistically significant. # means a significant difference from the CD group, * means a significant difference from the HFD group. Symbols used for significance levels are as follows: # or * for *p* < 0.05, ## or ** for *p* < 0.01, ### or *** for *p* < 0.001, and #### or **** for *p* < 0.0001; average ± standard deviation. The black circles in the figure represent data from individual subjects in each group.

## 3. Results

### 3.1. Mouse Body Weight and Blood Glucose Levels

Body weight is a fundamental index for monitoring physiological changes in animals. In this study, the body weights of the mice were recorded weekly throughout the experiment. Finally, the body weights at 12 weeks were plotted on a line graph to clearly illustrate the dynamic changes in body weight.

**Figure 1:**
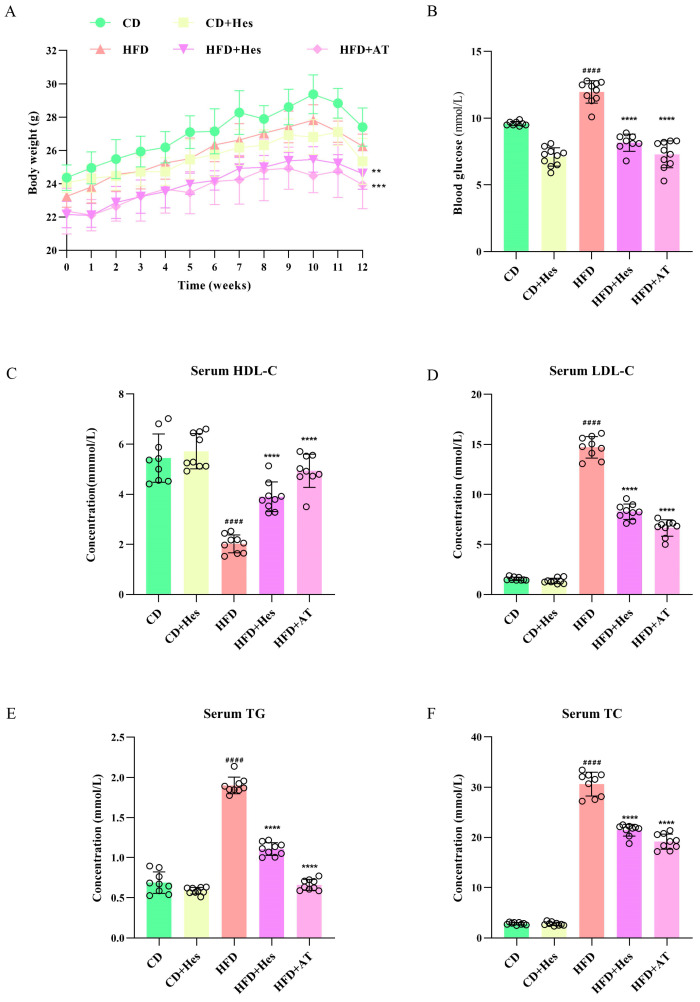
Effects of hesperidin on physiological health and dyslipidaemia in mice. (**A**) Body weights of mice in each group recorded at 0–12 weeks of normal feeding; (**B**) Fasting blood glucose levels in mice; (**C**) Serum high-density lipoprotein levels in mice; (**D**) Serum low-density lipoprotein levels in mice; (**E**) Serum triglyceride levels in mice; (**F**) Serum total cholesterol levels in mice. Data are expressed as the mean ± SD (*n* = 9). The statistical analysis was performed using one-way ANOVA followed by Tukey’s test. Different letters indicate significant differences (*p* < 0.05). # means a significant difference from the CD group, * means a significant difference from the HFD group. Symbols used for significance levels are as follows: ** for *p* < 0.01, *** for *p* < 0.001, and #### or **** for *p* < 0.0001.

Studies have shown that hyperglycaemia is a strong risk marker for atherosclerosis [17,18]. Therefore, it is also necessary to test the level of fasting blood glucose in mice. Before the study, mice underwent a 12-h water fast to measure and record the fasting blood glucose levels in each group of mice. As depicted in Figure 1B, fasting blood glucose was significantly higher in the high-fat diet induced model group (11.97 ± 1.2 mmol/L) (## *p* < 0.01) than in the control group (9.6 ± 0.8 mmol/L), whereas the hesperidin intervention group (8.16 ± 0.7 mmol/L) (** *p* < 0.01) showed a statistically significant improvement compared with the atorvastatin group (7.29 ± 0.6 mmol/L) (** *p* < 0.01). In conclusion, hesperidin has the ability to alleviate high-fat diet induced atherosclerosis.

### 3.2. Hesperidin Treatment Reduces Dyslipidaemia in Atherosclerotic Mice

Atherosclerosis is primarily caused by dyslipidemia, also known as hyperlipidemia [19]. Hyperlipidemia specifically refers to four abnormalities in plasma lipid levels, including high levels of TC, TG, LDL-C and low levels of HDL-C [20]. Serum was collected for biochemical analysis, and the results are shown in Figure 1C–F. In the CD group, the TC level was approximately 2.85 ± 0.24, the TG level was around 0.69 ± 0.13, the LDL-C level was about 1.57 ± 0.18, and the HDL-C level was approximately 5.45 ± 0.96. In the atherosclerosis model mice, the levels of serum TC, TG, and LDL-C were significantly increased to approximately 30.64 ± 2.37, 1.91 ± 0.10, and 14.72 ± 1.08, respectively. However, the serum HDL-C level was significantly decreased to 2.02 ± 0.36. In addition, experimental data based on HFD + Hes showed that hesperidin intervention significantly reduced serum TC and LDL-C levels (*** *p* < 0.001) and upregulated HDL-C (##, ** *p* < 0.01). The data from the HFD + AT groups showed that atorvastatin was also able to significantly optimise these lipid-related parameters. Taken together, these data suggest that hesperidin attenuates related parameters of atherosclerosis in mice.

### 3.3. Hesperidin Treatment Improves Lipid Deposition in Atherosclerotic Mice

**Figure 2:**
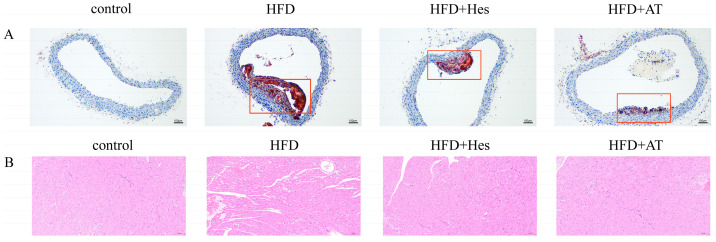
Hesperidin administration improves lipid deposition in high-fat, high-cholesterol diet-induced atherosclerotic mice (100×). (**A**) Cross section of mouse aorta stained with oil red O; (**B**) H&E staining of mouse aortic root.

Studies have shown that feeding mice a high-fat, high-cholesterol diet can mimic experimental atherosclerosis [21]. After 12 weeks of treatment, the hearts and aortas of the mice were examined. The results showed significant thickening of the arterial wall, widening of the intimal space, cholesterol and lipid deposition, and aortic calcification in the atherosclerotic mice, the area of the patch reached 0.212 μm. Treatment with hesperidin or atorvastatin for 11 weeks showed improvements in the arterial wall thickness and reduction in cholesterol and lipid deposition, the area of the patch decreased to 0.087 μm and 0.063 μm, respectively. Hesperidin also led to a dose-dependent reduction in lipid deposition and improvement in the aorta’s structure. Atorvastatin had the most significant effect in reducing lesions in the atherosclerotic mice. This suggests that hesperidin administration was able to partially reduce lipid deposition in atherosclerotic mice. Additionally, H&E staining of the heart root revealed that the atherosclerotic lesion area in the model mice was significantly larger than that in the control group (Figure 2B). However, hesperidin dose-dependently decreased the plaque size in the atherosclerotic mice, and the positive drug atorvastatin also significantly improved the atherosclerotic plaque area in the heart root. This further confirmed the mitigating effect of hesperidin administration on lipid deposition in atherosclerotic mice.

### 3.4. Hesperidin Administration Ameliorates Liver Injury in Atherosclerotic Mice

**Figure 3:**
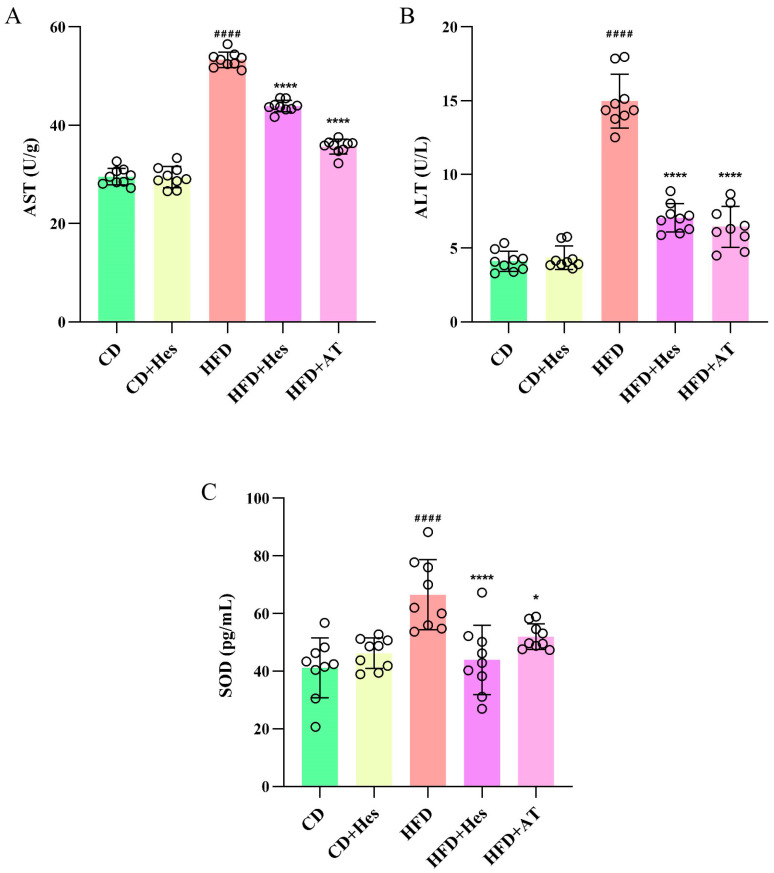
Hesperidin administration ameliorates liver injury in atherosclerotic mice induced by a high-fat, high-cholesterol diet. (**A**) Aspartate aminotransferase level in mouse liver; (**B**) Alanine aminotransferase level in mouse liver; (**C**) Superoxide dismutase level in mouse liver. Data are expressed as the mean ± SD (*n* = 9). The statistical analysis was performed using one-way ANOVA followed by Tukey’s test. Different letters indicate significant differences (*p* < 0.05). # means a significant difference from the CD group, * means a significant difference from the HFD group. Symbols used for significance levels are as follows: * for *p* < 0.05, and #### or **** for *p* < 0.0001.

AST and ALT levels can indicate inflammation and liver injury, which are markers of atherosclerosis [22]. The levels were measured using ELISA, and the results are shown in Figure 3A,B. In the model group, AST and ALT levels were significantly higher (## *p* < 0.01) at 53.31 ± 1.60 and 14.97 ± 1.82 compared to CD mice at 29.55 ± 1.68 and 4.11 ± 0.69. Additionally, the levels of AST and ALT in the group supplemented with hesperidin were approximately 88 ± 1.171 and 7.06 ± 0.96 (** *p* < 0.01), respectively, while those in the HFD + AT group were around 35.64 ± 1.51 and 6.45 ± 1.40 (** *p* < 0.01). This indicates a significant reduction in AST and ALT levels after hesperidin-supplemented gavage, although slightly less effective compared to the positive drug group. The observed anti-inflammatory properties of hesperidin may further alleviate liver injury in mouse models of atherosclerosis.

Furthermore, SOD is a key antioxidant enzyme in the liver, neutralizing superoxide radicals to protect hepatocytes. It acts synergistically with other antioxidants to preserve hepatic homeostasis, and its expression is significantly upregulated under oxidative stress to alleviate related damage. This return to normal SOD levels indicates restored redox balance. SOD levels in liver samples showed that the SOD level in mice of the CD group was 41.15 ± 10.36, and the SOD level in mice of the atherosclerosis model group was significantly higher compared to the CD group (** *p* < 0.01), up to 66.52 ± 12.15. The level in the mice in the hesperidin-treated group was significantly lower than that in the HFD group (Figure 3C). The increased SOD activity in the HFD group represents a compensatory—yet insufficient—response to severe oxidative stress. In contrast, the decreased SOD activity in the HFD + Hes group does not reflect impaired antioxidant capacity. Instead, hesperidin treatment reduced oxidative stress, diminishing the need for SOD upregulation. This further demonstrates that hesperidin has a reversing effect on atherosclerosis-induced liver injury.

### 3.5. Administration of Hesperidin Improves Levels of Atherosclerosis in Mice in the Model Group

**Figure 4:**
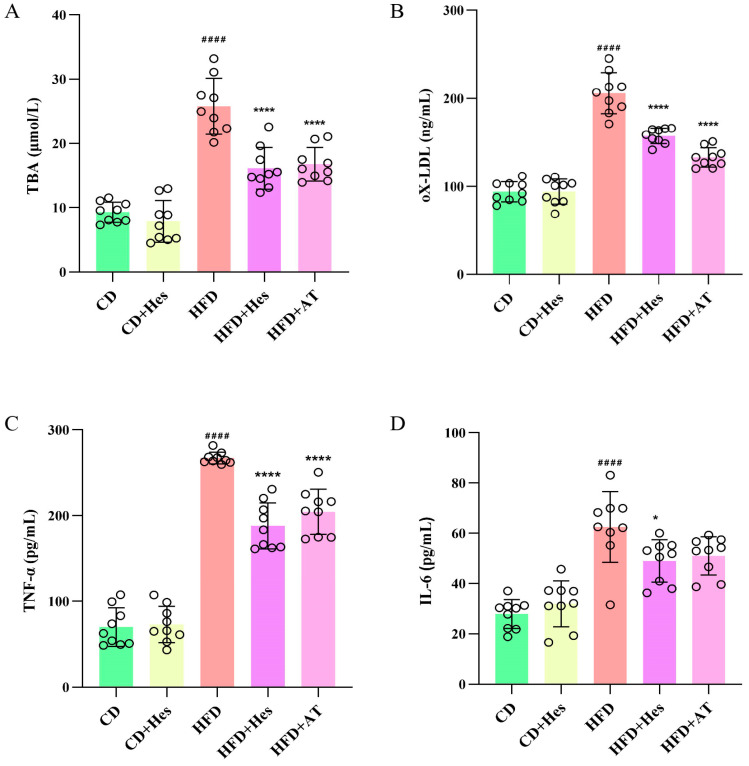
Hesperidin treatment alleviates atherosclerosis markers induced by a high-fat, high-cholesterol diet in mice of the model group. (**A**) Levels of total bile acids in mice; (**B**) Levels of oxidatively modified LDL in mice; (**C**) Levels of tumour necrosis factor-α in mice; (**D**) Levels of interleukin-6 in mice. Data are expressed as the mean ± SD (*n* = 9). The statistical analysis was performed using one-way ANOVA followed by Tukey’s test. Different letters indicate significant differences (*p* < 0.05). # means a significant difference from the CD group, * means a significant difference from the HFD group. Symbols used for significance levels are as follows: * for *p* < 0.05, and #### or **** for *p* < 0.0001.

The conversion of cholesterol to TBA plays a key role in maintaining cholesterol homeostasis and preventing the accumulation of cholesterol, triglycerides and toxic metabolites [23], and TBA has been associated with cardiovascular disease in animals and humans [24,25]. Therefore, the determination of TBA levels in mice is necessary for atherosclerosis-related experiments. Liver tissue samples (or liver homogenates) can be used for ELISA to determine the bile acid content in different groups of mice. The results indicated that the control group had a level of 9.29 ± 1.56, while the model mouse group showed a significant increase to 25.79 ± 4.35 compared to the control group (## *p* < 0.01). In contrast, the TBA level in the HFD + Hes mice decreased significantly to 16.14 ± 3.243 compared to the model group (** *p* < 0.01) (Figure 4A). Additionally, the TBA level in the HFD + AT mice was approximately 16.78 ± 2.61, also showing a significant decrease compared to the model group (** *p* < 0.01). This study showed that hesperidin had a beneficial effect on the atherosclerotic condition of the mice, and the impact was similar to that of the positive drug.

oX-LDL is strongly associated with disease severity in patients with atherosclerosis [26], is a major factor in foam cell formation and is also involved in the formation of atherosclerotic plaques [27]. Therefore, serum was used as the sample for the determination of oxidatively modified LDL content in mice. As shown in Figure 4B, the oX-LDL level of mice in the model group was higher (205.89 ± 23.38) compared to the normal group (94.02 ± 11.42) (## *p* < 0.01)and the administered group (157.4 ± 8.64), whereas the oX-LDL level of HFD + Hes mice was significantly reduced after hesperidin supplementation (** *p* < 0.01). In addition, the blood of the mice was tested for inflammatory factors, and the results showed that the increase in both TNF-α and IL-6 in atherosclerotic mice was suppressed after hesperidin gavage supplementation (Figure 4C,D). Whether or not inflammatory factors are elevated is also an important factor in detecting atherosclerosis, these findings further substantiate the antioxidant and anti-inflammatory properties of hesperidin, demonstrating its efficacy in mitigating atherosclerosis in high-fat/high-cholesterol diet-induced mouse models.

### 3.6. Hesperidin Administration Improves the Gut Microbiota in Atherosclerotic Mice

#### 3.6.1. Changes in the Intestinal Flora

**Figure 5:**
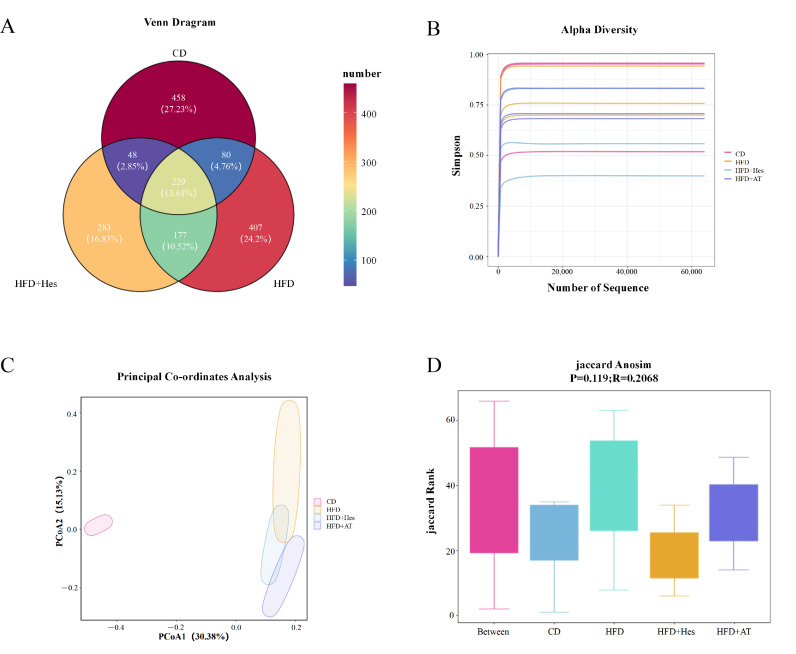
Effects of hesperidin on the intestinal flora of mice with atherosclerosis induced by high-fat, highcholesterol diet. (**A**) Number of ASVs of intestinal flora in each group; (**B**) Dilution curves of intestinal flora in each group of mice; (**C**) Principal component analysis of intestinal flora in each group of mice; (**D**) Jaccard Anosim analysis of intestinal flora in each group of mice.

Previous studies have shown that the gut microbiome is closely associated with the progression of atherosclerosis [28]. We analyzed the fecal microbiota of ApoE^−/−^ mice with or without hesperidin supplementation using 16S rRNA gene sequencing (Table 1 and Table 2). Based on 16S rRNA sequencing of fecal samples from ApoE^−/−^ mice, hesperidin supplementation was associated with compositional changes in the gut microbiota. Venn analysis revealed group-specific Amplicon Sequence Variants (ASVs), with 458, 407, and 283 unique ASVs in the control, model (HFD), and hesperidin-treated (HFD + Hes) groups, respectively (Figure 5A). The dilution curves reached a plateau, indicating sufficient sequencing depth and stable estimates of alpha diversity (Figure 5B). Although statistically non-significant (*p* > 0.05), the Jaccard Anosim analysis (Figure 5D) indicated certain compositional divergences in the gut flora across the CD, HFD, HFD + Hes, and HFD + AT groups. This was supported by PCoA, which showed clear separation between control (CD) and high-fat diet (HFD) groups, and partial yet distinguishable clustering of the HFD + Hes and HFD + AT groups relative to the HFD group (Figure 5C).

#### 3.6.2. Species Composition and Differential Analysis in the Gut Flora

**Figure 6:**
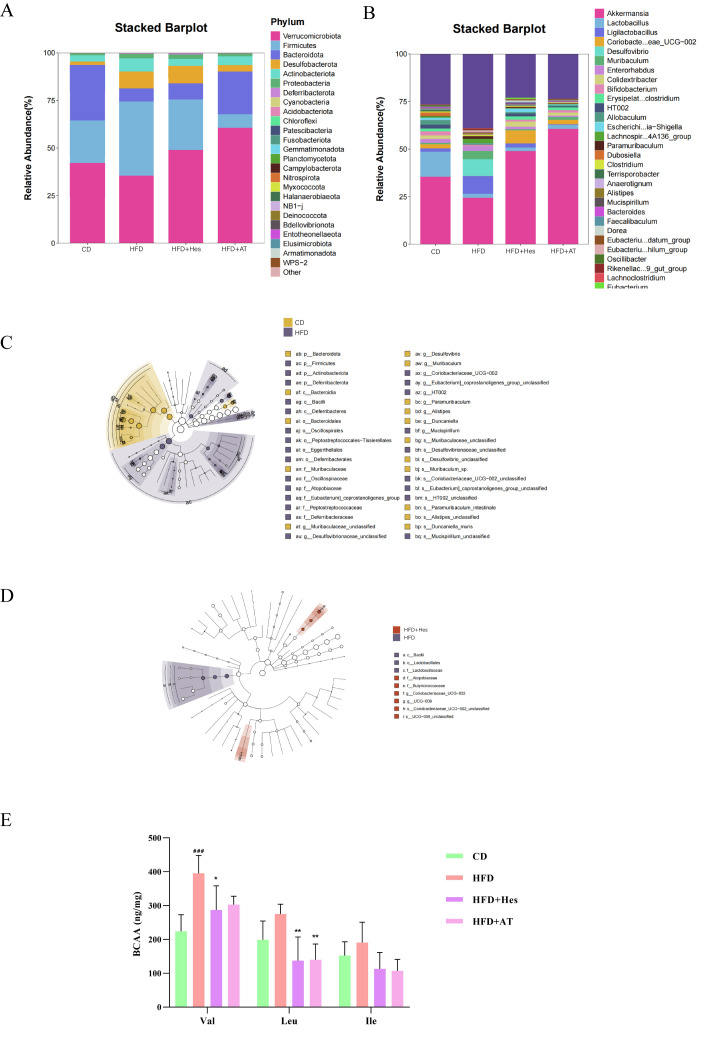
Effects of hesperidin on the composition and differential analysis of intestinal flora species and branched-chain amino acid content in mice. (**A**) Composition at the level of intestinal flora phylum in mice; (**B**) Composition at the level of intestinal flora genus in mice; (**C**) Species differences between the model and normal treated groups; (**D**) Species differences between the model and hesperidin supplemented treated groups; (**E**) Branched-chain amino acid content in mice of each group. The statistical analysis was performed using one-way ANOVA followed by Tukey’s test. Different letters indicate significant differences (*p* < 0.05). # means a significant difference from the CD group, * means a significant difference from the HFD group. Symbols used for significance levels are as follows: * for *p* < 0.05, ** for *p* < 0.01, ### for *p* < 0.001.

At the phylum level, 16S rRNA sequencing revealed that the gut microbiota of all experimental mice was predominantly composed of *Verrucomicrobia*, *Firmicutes*, *Bacteroidota*, *Actinobacteriota*, *Desulfobacterota*, and *Proteobacteria* (Figure 6A). Compared to the control group, mice in the atherosclerosis model (HFD) group exhibited a marked increase in the relative abundance of *Firmicutes*, accompanied by significant decreases in *Verrucomicrobia* and *Bacteroidota*. Supplementation with hesperidin (HFD + Hes) and the reference treatment (HFD + AT) partially mitigated these diet-induced alterations, resulting in a reduction in Firmicutes and an increase in both *Verrucomicrobia* and *Bacteroidota* relative to the HFD group. Further analysis at the genus level (Figure 6B) indicated that the HFD model group displayed a decreased relative abundance of *Akkermansia* and *Lactobacillus*, alongside an increase in *Ligilactobacillus* compared to controls.

LEfSe analysis with an LDA score threshold of >2.0 and a *p*-value of <0.05 revealed statistically significant differences in gut microbial composition across the experimental groups. As visually summarized in Figure 6C,D, specific bacterial taxa demonstrated distinct enrichment patterns: the control group showed higher relative abundances of p_Bacteroidota, c_Bacteroidia, o_Bacteroidales, f_Muribaculaceae, and s_Paramuribaculum_intestinale. In contrast, the atherosclerosis model (HFD) group exhibited marked enrichment of p_Firmicutes, c_Bacilli, and p_Actinobacteriota, with c_Bacilli, o_Lactobacillales, and f_Lactobacillaceae being particularly predominant. Notably, hesperidin supplementation (HFD + Hes group) resulted in a distinct microbial signature characterized by a significant increase in f_Atopobiaceae, g_Coriobacteriaceae_UCG-002, and s_Coriobacteriaceae_UCG-002_unclassified, indicating a shift in microbial community structure following treatment.

### 3.7. Hesperidin Administration Improves BCAA Levels in Atherosclerotic Mice

Essential amino acids, including valine (Val), leucine (Leu), and isoleucine (Ile), are known as BCAA [29]. They serve as direct and indirect nutritional signals [30]. Several studies have shown that BCAA levels in the body play an important role in the dietary composition and metabolism of patients with cardiovascular disease [31]. Elevated levels of BCAA indicate an increased likelihood of developing cardiovascular disease. Through the detection of BCAA content in mouse faeces, it was found that the content of Val, Leu and Ile in mice of the HFD group was 395.57, 275.4, and 190.8, respectively. These levels were significantly higher than those of mice in the CD group, which were 223.64, 199.2, and 152.3. After treatment with hesperidin, the content of Val, Leu and Ile in mice of the HFD + Hes group was reduced to 287.09, 137.55, and 113.03, respectively (Figure 6E). It can be seen that the levels of Val, Leu and Ile in the faeces of the model group were reduced by 27.4%, 50.1% and 40.8%, respectively, after the hesperidin intervention (*p* < 0.05).

Further correlation analysis indicated that the reduction in BCAA levels was closely associated with hesperidin-induced modulation of the gut microbiota. Specific bacterial taxa affected by hesperidin were significantly correlated with changes in Val, Leu, and Ile concentrations, suggesting that the microbiota plays a key role in BCAA metabolism. This interaction forms a coherent metabolism–flora chain of evidence supporting the mechanism by which hesperidin exerts its effects. The results of the BCAA and 16S rRNA analyses showed that hesperidin could regulate the intestinal flora and affect BCAA metabolism to alleviate the levels of atherosclerosis induced by a high-fat and high-cholesterol diet.

## 4. Discussion

AS is a major underlying cause of cardiovascular diseases, contributing significantly to global morbidity and mortality. It affects about 422.7 million people and accounts for 31% of deaths worldwide [32]. Although current pharmacological treatments like statins are widely used to manage lipid abnormalities in AS, they carry risks of side effects such as hepatotoxicity and acute renal failure [33]. This has spurred interest in identifying safer natural anti-atherosclerotic compounds. For example, hesperidin, abundant in Chen Pi, exhibits anti-inflammatory and antioxidant properties, and has shown potential in alleviating atherosclerosis [34]. In this study, we used ApoE^−/−^ mice as an AS model to investigate the mechanisms behind hesperidin’s protective effects.

In this study, serum TG and TC levels were significantly reduced in hesperidin-treated mice, which may be attributed to its regulatory effects on gut microbiota and the metabolite BCAA [35]. Furthermore, hesperidin supplementation significantly decreased LDL-C levels and increased HDL-C levels in AS model mice. Similarly, Luo et al. [36] reported that hesperidin inhibited lipid accumulation and ameliorated dyslipidemia by suppressing cellular pyroptosis. These findings suggest that hesperidin may alleviate AS by reversing dyslipidemia and reducing inflammation. Additionally, hesperidin significantly reduced hepatic TBA levels, indicating that gut microbiota may also influence liver TBA levels and contribute to improved gut health [37].

In the model group, levels of TNF-α and IL-6 were elevated but significantly reduced by hesperidin treatment, indicating its anti-inflammatory role in attenuating atherosclerosis. Consistent with this, Hanchang et al. [38] showed that hesperidin modulates Bcl-2 proteins and inhibits hyperglycemia-induced splenocyte apoptosis. Additionally, hesperidin increased hepatic SOD activity, enhancing endogenous antioxidant capacity. Since ROS-driven LDL oxidation accelerates atherogenesis, SOD plays a key role in limiting ox-LDL formation [39]. Notably, hesperidin supplementation markedly reduced ox-LDL levels. This antioxidant effect further supports its anti-atherogenic potential, corroborating findings by Bhargava et al. [40] that hesperidin alleviates oxidative stress via the PPAR-γ pathway. Together, these mechanisms underline the dual protective role of hesperidin through both inflammatory suppression and oxidative defense.

Research demonstrates that hesperidin supplementation induces structural changes in the gut microbial community in a mouse model of atherosclerosis, reflected by distinct ASVs and PCoA clustering, along with significant enrichment of taxa such as Coriobacteriaceae per LEfSe analysis, suggesting a prebiotic-like effect [41]. These shifts include altered abundances of bacterial phyla (*Firmicutes*, *Bacteroidota*) and genera (*Akkermansia*, *Lactobacillus*), partially reversing high-fat diet-induced dysbiosis linked to cardiovascular pathogenesis. Although these modifications may influence microbial functions in nutrient and bile acid metabolism [42] and involve axes such as gut-liver-heart [43], the lack of significant overall community similarity indicates that hesperidin’s effects are subtle and secondary to dietary influence.

After ingestion, hesperidin reaches the intestine and modulates gut microbiota, which plays a critical role in atherosclerosis development [44,45,46]. Gut dysbiosis is associated with chronic inflammation and dyslipidemia, exacerbating disease progression [47]. In this study, hesperidin increased the abundance of Bacteroidales, which was reduced in atherosclerotic mice, thereby influencing gut metabolic activity and leading to reduced hepatic BCAA levels (Val, Leu; *p* < 0.05). Lower BCAAs may ameliorate inflammation and dyslipidemia [48,49], potentially attenuating atherosclerosis. Additionally, microbial modulation might enhance hepatic antioxidant response [50]. These results uncover a novel “gut microbiota–BCAA” pathway mediating the anti-atherosclerotic effects of hesperidin.

Finally, the influence of gut flora and BCAAs on atherosclerotic lesions was corroborated by pathological analysis. H&E-stained sections showed that hesperidin supplementation reduced the aortic lesion area in ApoE^−/−^ mice fed a high-fat, high-cholesterol diet. Oil Red O staining of aortic root cross-sections revealed reduced foam cell formation and improved cell arrangement in the hesperidin group. These results demonstrate that hesperidin reduces atherosclerotic plaques and inflammatory cells, thereby alleviating AS.

## 5. Conclusions

In summary, this study demonstrates that hesperidin supplementation alleviates atherosclerosis in ApoE^−/−^ mice fed a high-fat, high-cholesterol diet. It improved multiple cardiometabolic parameters, including hyperglycemia, dyslipidemia, inflammation, and oxidative stress, and reduced atherosclerotic plaque burden. These benefits were associated with a restructuring of the gut microbiota, characterized by an increase in beneficial bacteria and enhanced microbial catabolism of BCAAs. Our data suggest that the attenuation of atherosclerosis by hesperidin is associated with modulation of the gut microbiota–BCAA–host axis and a reduction in circulating levels of these atherogenic metabolites. These findings support the potential of hesperidin as a dietary intervention targeting the gut microbiome. However, the results demonstrate correlation, not causation. Future studies using fecal microbiota transplantation or antibiotic depletion in ApoE^−/−^ mice are required to establish a causal role for the gut microbiota in this mechanism.

## Figures and Tables

**Table 1:** 16S rRNA Date.

Sample	Raw_Reads	Raw_Bases	Valid_Tags	Valid_Bases	Valid%	Q20%	Q30%	GC%
CD	82,502	41.25 M	77,365	32.26 M	93.76	98.11	94.15	54.86
HFD	83,714	41.86 M	77,985	32.34 M	93.06	98	93.86	53.89
HFD + Hes	81,481	40.74 M	76,150	31.32 M	93.45	98.04	93.95	54.53

**Table 2:** Alpha diversity index of each sample.

Sample	Observed_Species	Shannon	Simpson	Chao1	Goods_Coverage	Pielou_e	Ace
CD	400	4.82	0.81	401.25	1	0.57	402.37
HFD	236	3.47	0.76	236.16	1	0.44	236.76
HFD + Hes	354	3.94	0.74	354.11	1	0.47	354.87
HFD + AT	355	3.24	0.60	355.55	1	0.38	356.90

## Data Availability

The original contributions presented in the study are included in the article, further inquiries can be directed to the corresponding authors.

## Abbreviations

The following abbreviations are used in this manuscript:

ApoE^−/−^ mice	apolipoprotein E-deficiency mice
AS	atherosclerosis
BCAA	branched-chain amino acid
CVD	cardiovascular disease
CMC-Na	Sodium carboxymethyl cellulose
TC	total cholesterol
TG	triglycerides
HDL-C	high-density lipoprotein cholesterol
LDL-C	low-density lipoprotein cholesterol
AST	aspartate aminotransferase
ALT	alanine aminotransferase
TBA	total bile acids
ox-LDL	oxidized low-density lipoprotein
IL-6	interleukin-6
TNF-α	tumor necrosis factor-alpha
